# Influence of psychological inflexibility and mindfulness on hypersexuality and sexual satisfaction in a Spanish sample

**DOI:** 10.3389/fpsyg.2023.1182222

**Published:** 2023-07-20

**Authors:** Marta Ortega-Otero, Francisco Montesinos, Luka Charrabe

**Affiliations:** Department of Psychology, Faculty of Biomedical and Health Sciences, Universidad Europea de Madrid, Madrid, Spain

**Keywords:** body awareness, bodily dissociation, cognitive fusion, hypersexuality, mindfulness, psychological flexibility, sexual satisfaction

## Abstract

Sexual satisfaction is tremendously relevant to wellbeing and quality of life. Inversely, hypersexuality may increase the risk of psychological distress, unprotected sex, and marital problems. This study attempts to explore the sociodemographic variables related to hypersexuality and the relationship between hypersexuality and sexual dissatisfaction with psychological inflexibility, cognitive fusion, body awareness, bodily dissociation, and mindfulness skills in the Spanish population. The sample was made up of 530 participants between 18 and 67 years of age. In total, 5.5% displayed hypersexuality, more likely in males, lesbians and gay men, singles, and students. An online survey was conducted including standardized questionnaires as follows: AAQ-II, CFQ, SBC, MAAS, NSSS-S, and HBI. Significant moderate positive correlations were found between hypersexuality and psychological inflexibility, cognitive fusion, and bodily dissociation, and an inverse moderate correlation with mindfulness skills. Moderate negative correlations were found between sexual satisfaction and bodily dissociation. Linear regression showed psychological inflexibility was the variable with the most weight on hypersexuality, while bodily dissociation was the variable with the most influence on sexual dissatisfaction. This study suggests lesbians and gay men, males, and singles may show an increased vulnerability toward engaging in hypersexual behavior and provides justification for interventions focused on psychological inflexibility and mindfulness skills to treat hypersexuality and improve sexual satisfaction.

## 1. Introduction

### 1.1. Hypersexuality

Hypersexuality is the uninhibited or excessive expression of culturally adapted sexual conduct that leads to important adverse consequences (Kafka, 2010). It is also known as sexual compulsivity or sex addiction, and it includes a combination of excessive sexual desire plus a pathological incapacity to control it (Ballester-Arnal et al., 2019). Hypersexuality has been related to the male gender (Ballester-Arnal et al., 2013; Castro-Calvo et al., 2017), adulthood (Amamou et al., 2020), homosexuality (Daneback et al., 2005), lower educational levels (Kafka and Hennen, 1999), and child sexual abuse (Slavin et al., 2020). ICD-11 included the diagnosis of compulsive sexual behavior (CSB) as an impulse control disorder (World Health Organization, 2018). Recent studies estimate its occurrence in Spain to be at 10.12% for university students and at 7.81% for the general population (Castro-Calvo et al., 2020). Many risks of hypersexuality have been pointed out. It is linked to higher pornography use, frequency and compulsiveness of masturbation, a higher number of sexual partners, and impersonal sexual activity (Klein et al., 2014; Wordecha et al., 2018). It has also been related to unprotected sex and HIV-associated sexual risk behavior (Grov et al., 2010). Furthermore, hypersexuality may cause significant levels of psychological distress and impairment regarding partner relationships (Reid et al., 2010; Spenhoff et al., 2013). It may also be associated with shame, rumination (Reid et al., 2014a), anxiety and depression (Štulhofer et al., 2016; Castro-Calvo et al., 2020), and even suicidal behaviors in extreme cases (Chatzittofis et al., 2017). Hypersexuality has also been connected to sexual dissatisfaction (Wéry and Billieux, 2016; Dwulit and Rzymski, 2019; Kowalewska et al., 2019; Miner et al., 2019).

### 1.2. Sexual satisfaction

Sexual satisfaction has been defined as the subjective evaluation a person makes of his/her sexual relationships (Lawrance and Byers, 1995). It is associated with sexual assertiveness (Santos-Iglesias and Sierra, 2010), quality of intimate communication (Byers, 2005), satisfaction with body image (Higgins et al., 2011), frequency of sexual encounters (Terzera et al., 2020), and number of orgasms (Dyar et al., 2020). It has a very significant connection to marital satisfaction, which is necessary to create enduring and solid family ties (Ashdown et al., 2011; Yoo et al., 2014). It also contributes to the quality of life (Ventegodt, 1998) and prevents risky sexual behaviors (Hajivosough et al., 2012). In addition, studies have related sexual satisfaction to body awareness (van Kolthoorn, 2018; Sánchez-Sánchez et al., 2021) and psychological flexibility (PF) (Tapp, 2014).

### 1.3. Psychological inflexibility

When explaining the maintenance of hypersexual behaviors, these have been pointed as a way to regulate mood and stress (Wordecha et al., 2018). Specifically, hypersexual behaviors have been related to psychological inflexibility (PI) (Wetterneck et al., 2012). PI is defined as the “inability to persist or change in the service of long-term valued ends” (Hayes et al., 2006). PI refers to patterns of behavior regulated by six processes: (1) emotional avoidance, (2) cognitive fusion (CF), (3) attachment to the conceptualized self, (4) inaction, impulsivity, or avoidance persistence, (5) lack of values clarity, and (6) loss of flexible contact with the present (Hayes et al., 2013). PI has been related to the maintenance of diverse general psychological problems and disorders, such as work stress, pain, anxiety, depression, obsessive-compulsive disorders (Hayes et al., 2011), drug abuse (Páez and Montesinos, 2019), and to specific disorders, such as impulsivity (Zeliha, 2022), problematic internet and smartphone use (Kuru and Celenk, 2021; Liu et al., 2022), problematic internet pornography use (Wetterneck et al., 2012), and internet addiction (Hsieh et al., 2019). Although PI is a promising and innovative construct that is arousing considerable interest, some researchers have urged that it should be used with caution (Kollman et al., 2009) while others have provided evidence supporting PF as a unitary construct (Gloster et al., 2011) and its discriminant validity appears to be strong regardless of the instrument used to measure it (Ong et al., 2020).

Two of the processes included in PI, CF and loss of flexible contact with the present, have shown their specific relationship with emotion dysregulation and impulsive behaviors in different contexts (Cox et al., 2018; Jo and Yang, 2019; Barney, 2023), and it is worth exploring their relationship with hypersexuality and sexual satisfaction. CF has been defined as the tendency to believe the literal content of thoughts and emotions. It refers to excessive or inappropriate control of language, which has as its consequence a behavior that is inadequate within a given set of circumstances and is controlled by inflexible verbal rules rather than by its contingencies (Hayes et al., 2011). An example of CF is experiencing the thought “I'm going to fail” as if it were reality itself and behaving as if one had already failed. On the other hand, loss of flexible contact with the present can be considered synonymous with mindfulness and includes lack of body awareness (BA), which refers to attention focus on and awareness of internal body sensations (Mehling et al., 2009) and implies perceiving, recognizing, and being connected with corporal states, processes, and actions (Quezada-Berumen et al., 2014) and seems to be inversely related to hypersexuality (Selice and Morris, 2021). Mindfulness refers to focused, voluntary, and flexible attention and contact with ongoing experiences. Flexible attention to the now means choosing to pay attention here and now to one's own experiences (Hayes, 2020). Mindfulness training may be helpful in promoting better sexual functioning and satisfaction (Dove and Wiederman, 2000; Mayland, 2005).

Considering the interest in sexual satisfaction as a remarkable element of health and quality of life, and since concern about the risks associated with hypersexuality is increasing, a better understanding of sexual satisfaction and hypersexuality and the variables that could contribute to explaining them is necessary. This could help to innovate in the design of future psychological interventions for treating hypersexuality and improving sexual satisfaction. Many studies have highlighted the role of PI and its processes in the maintenance of a wide range of psychological disorders (Hayes, 2019), and recent research suggests the possibility of explaining hypersexuality as an inflexible way to cope with stress and discomfort through emotional avoidance and lack of contact with body sensations. In this context, this study aimed to explore the sociodemographic variables related to hypersexuality and the relationship between hypersexuality and sexual satisfaction with PI and other related processes, such as CF, BA, and mindfulness-related skills.

## 2. Materials and methods

### 2.1. Participants

The sample consisted of 530 participants aged between 18 and 67 years (M = 31.9; SD = 10.1). Most of them were women (76.6%), Spanish (89.1%), and resided in Madrid (61.5%). In total, 78.9% were heterosexual, 55.1% considered that they were not religious, and 74.5% considered that their political orientation was left-wing or center-left. A total of 84.3% were graduates, 49.2% were employees, and 28.7% were students. In total, 50.6% were single, and 46.4% were married or cohabiting. For those that had a partner, the average length of the relationship was of 7.6 years (SD = 8.4; range 0–42). For more detail on sociodemographic data, please refer to Table 1.

**Table 1:** Sociodemographic characteristics.

**Variables**	**Values**	**Percentage**
Gender	**Female**	76.6
	Male	23.4
Country of origin	**Spain**	89.1
	Latin America	8.87
	Rest of Europe	2.08
Place of residence	**Madrid**	61.5
	Other	38.5
Sexual orientation	**Heterosexual**	78.9
	Homosexual	4.5
	Bisexual	14.7
	Other/I don't know	1.9
Religious beliefs	**Not religious**	55.1
	Not very religious	37.2
	Quite/very religious	7.7
Political ideology	Right	6.0
	Center	18.5
	**Left**	74.5
Schooling	Primary and secondary studies	15.7
	**College graduate**	84.3
Occupation	Student	28.7
	Unemployed or retired	8.9
	Business owner or self-employed	13.2
	**Employee**	49.2
Civil status	**Single**	50.6
	Married	18.9
	Cohabiting	27.5
	Separated or divorced	3

### 2.2. Instruments

*Acceptance and Action Questionnaire-II* (AAQ-II) (Bond et al., 2011). It is a frequently used tool to assess experiential avoidance and psychological inflexibility. The Spanish version was adapted by Ruiz et al. (2013). It is made up of seven items that use a seven-point Likert-type scale (from “never true” to “always true”). Higher scores indicate higher levels of PI. A good internal consistency has been found (α = 0.74), similar to that of the original version (Barraca, 2004), as well as adequate construct, discriminant, and external validities (Ruiz et al., 2013). Cronbach's alpha of the AAQ-II in this study was 0.9.

*Cognitive Fusion Questionnaire* (CFQ) (Gillanders et al., 2014). It is a widely used questionnaire to specifically assess cognitive fusion. The Spanish version was published by Romero-Moreno et al. (2014). This instrument assesses CF. It is made up of seven items that use a seven-point Likert-type scale (from “never true” to “always true”). The higher the score in the questionnaire, the higher the tendency to believe the literal content of private events. The internal consistency in the Spanish version was 0.87 (Romero-Moreno et al., 2014). Cronbach's alpha in the present sample is 0.93.

*The Scale of Body Connection* (SBC) (Price and Thompson, 2007). The Spanish version was adapted by Quezada-Berumen et al. (2014). It is a 20-item, 7-point Likert-type scale (from “not at all/never” to “all the time”). This instrument includes two subscales that assess BA (body awareness: the capacity to be aware of body states, processes, and actions, paying attention to the internal experience of the body) and BD (bodily dissociation: avoidance of body experiences). Cronbach's alpha in the Spanish validation was 0.86 for the BA subscale and 0.62 for the BD subscale (Quezada-Berumen et al., 2014). Cronbach's alpha in the present sample is 0.76.

*Mindful Attention Awareness Scale* (MAAS) (Brown and Ryan, 2003). The Spanish version was adapted by Soler et al. (2012). It is a 15-item 6-point scale (from “almost always” to “almost never”). It assesses mindfulness skills. Higher scores indicate a higher degree of attention to the present moment. The Spanish version showed a high internal consistency (α = 0.89; Soler et al., 2012). Cronbach's alpha of the MAAS in this study was 0.89.

*The New Sexual Satisfaction Scale-short form* (NSSS-S) (Štulhofer et al., 2010). The Spanish version was published by Strizzi et al. (2016). It is a 12-item, 7-point Likert-type scale (from “not at all satisfied” to “extremely satisfied”). It assesses sexual satisfaction. The Spanish version displayed adequate internal reliability (α = 0.92; Strizzi et al., 2016). Cronbach's alpha of the NSSS-S in this study was 0.9.

*Hypersexual Behavior Inventory* (HBI) (Reid et al., 2011). The Spanish version was published by Ballester-Arnal et al. (2019). It is a 19-item, seven-point Likert-type scale (from “never” to “many times”). The inventory measures three dimensions of hypersexuality: coping (display of sexual behavior in response to dysphoric moods), control (difficulty in managing impulses and other sexual behaviors), and consequences (persistence of sexual behaviors despite the risks they convey). The HBI scores range between 19 and 95. García-Barba et al. (2020) consider the presence of a problem of hypersexuality when the score is equal to or higher than 53. Bothe et al. (2019) confirmed that the instrument might be applied to non-clinical populations. Ballester-Arnal et al. (2019) obtained a reliability of in-between 0.89 and 0.96. In their study, García-Barba et al. (2020) concluded that the reliability for these three factors is in-between 0.92 and 0.93 and found α = 0.96 for the general scale. Cronbach's alpha of the HBI in this study was 0.92.

*The sociodemographic questionnaire* included age, gender, sexual orientation, country of origin, place of residence, religious beliefs, political ideology, schooling, occupation, and civil status.

### 2.3. Procedure

An anonymous online questionnaire was designed using Google Forms. It included informed consent, sociodemographic data, and the previously mentioned scales. The questionnaire was administered between February and April 2021, after obtaining the approval of the ethics committee of the Universidad Europea de Madrid. The questionnaire was rolled out using the snowball method. The study was mainly disseminated through social networks. Participation was voluntary. The anonymity of participants was guaranteed, as was the confidentiality of the information provided. Participants were asked to complete the questionnaire in a private place with enough intimacy. The inclusion criteria were (1) being of legal age, (2) being Spanish-speaking, and (3) having engaged in at least one sexual encounter within the last year.

### 2.4. Data analysis

Descriptive, correlational (Pearson's and linear regression), and mean difference (*t*-test) analyses were performed using SPSS v19 to examine the sample distribution, the means and standard deviations, and the interactions between the studied variables. Due to the absence of normality and homoscedasticity, the Kruskal–Wallis test was selected to compare means of sexual satisfaction and hypersexuality in the function of the studied sociodemographic variables. Linear regression analysis was performed to examine the role of PI, CF, body awareness, bodily dissociation, and mindfulness skills in predicting participant's level of hypersexuality and sexual dissatisfaction.

## 3. Results

### 3.1. Descriptive analysis

The degree of hypersexuality within the sample was low (M = 29.97; SD = 11.67, range: 19–95); being slightly higher in males (M = 32.9; SD = 13.2) in comparison with women (M = 29.07; SD = 11). According to the criteria established by García-Barba et al. (2020), 5.5% of the sample displays clinically relevant levels of hypersexuality. Out of the 29 persons with hypersexuality, 65.5% are women, 79.3% are heterosexual, 93.1% are students, and the mean age is 30.3. The means and standard deviations of all the studied variables can be found in Table 2.

**Table 2:** Means and standard deviations.

	**Minimum**	**Maximum**	**Mean**	**SD**
Sexual satisfaction	12	60	44.9	8.9
Hypersexuality	19	95	29.97	11.67
Psychological inflexibility	7	49	21.8	9.3
Cognitive fusion	7	49	24.7	10.1
Body awareness	0	48	31.8	7.2
Bodily dissociation	0	30	9.5	4.8
Mindfulness skills	1.3	6	3.9	0.9

The analysis of differences in hypersexuality showed differences relative to gender (H = 10.93, *p* = 0.001), reflecting differences between men and women (U = 20.23, *p* = 0.001) in a way that gender accounts for 1.9% of the variance in hypersexuality and that men score higher on hypersexuality (M = 32.9) than women (M = 29.1). Moreover, there were differences for the partner cohabiting variable (H = 57.98, *p* = 0.00) showing differences among those who lived on their own but had a partner and those who did not have a partner (U = 7113, *p* = 0.00), with those who had a partner displaying lower levels of hypersexuality. Different levels of hypersexuality were also found between those who cohabited with their partner and those who did not have a partner (U = 8885, *p* = 0.00), with those cohabiting with a partner showing lower levels of hypersexuality. Hence, the partner cohabitation variable accounts for 8.7% of the variance within hypersexuality. Furthermore, the analysis showed that those who scored the highest in hypersexuality are those who did not have a partner (M = 35.55) in comparison with those that did have a partner (M = 28.11). On the other hand, Tables 3, 4 present the paired comparisons, which showed statistically significant differences (H = 17.88, *p* = 0.00) between heterosexuals and bisexuals (U = 12203, *p* = 0.00) so that sexual orientation accounts for 1.5% of the variance within hypersexuality and that lesbians and gay men display higher levels (M = 34.25) than bisexuals (M = 32.31) and heterosexuals (M = 29.29). Finally, concerning the occupation variable, the paired comparisons showed statistically significant differences (H = 16.33, *p* = 0.003) between students and businesspersons or self-employed workers (U = 3974, *p* = 0.002) with occupation accounting for 1.7% of the variance in hypersexuality. It is concluded that students score higher (M = 31.84) than businesspersons or self-employed workers (M = 28). For more details, see Tables 3, 4.

**Table 3:** Kruskal–Wallis values for sexual satisfaction by sociodemographic variable.

	**Sexual satisfaction**		
	**H**	**Bilateral *p*-value**	**eta^2^**
Gender	2.344	0.126 (*p >* α)	-
**Couple**	**30.578**	**0.000 (*****p** **<*** α)^**^	**0.054**
Schooling	4.552	0.103 (*p >* α)	-
Ideology	3.972	0.410 (*p >* α)	-
Sexual orientation	3.025	0.388 (*p >* α)	-
Religion	3.514	0.476 (*p >* α)	-
Occupation	7.385	0.117 (*p >* α)	-

**Table 4:** Kruskal–Wallis values for hypersexuality by sociodemographic variable.

	**Hypersexuality**		
	**H**	**Bilateral *p*-value**	**eta^2^**
**Gender**	**10.893**	**0.001 (*****p** **<*** α)^**^	**0.019**
**Couple**	**56.980**	**0.000 (*****p** **<*** α)^**^	**0.087**
Schooling	3.130	0.209 (*p >* α)	-
Ideology	4.496	0.343 (*p >* α)	-
**Sexual orientation**	**17.888**	**0.000 (*****p** **<*** α)^**^	**0.015**
Religion	3.446	0.486 (*p >* α)	-
**Occupation**	**16.339**	**0.003 (*****p** **<*** α)^**^	**0.017**

### 3.2. Correlational analysis

As can be seen in Table 5, hypersexuality showed a significant moderate positive correlation with PI (*r* = 0.393, *p* < 0.01), CF (*r* = 0.364, *p* < 0.01), and BD (*r* = 0.329, *p* < 0.01), a moderate inverse correlation with mindfulness skills (*r* = −0.365, *p* < 0.01), and a low inverse correlation with sexual satisfaction (*r* =−0.165, *p* < 0.01). PI showed a significant high direct correlation with CF (*r* = 0.825, *p* < 0.01) and BD (*r* = 0.553, *p* < 0.01) and a moderate inverse correlation with mindfulness skills (*r* = −0.463, *p* < 0.01). Similarly, CF showed a significant strong direct correlation with BD (*r* = 0.506, *p* < 0.01) and a moderate inverse correlation with mindfulness (*r* = −0.460, *p* < 0.01). Sexual satisfaction showed a significantly low direct correlation with BA (*r* = 0.126, *p* < 0.01) and mindfulness-related skills (*r* = 0.288, *p* < 0.01) and a negative low or moderate correlation with PI (*r* = −0.273, *p* < 0.01), CF (*r* = −0.221, *p* < 0.01), and BD (*r* = —.378, *p* < 0.01).

**Table 5:** Correlations between variables.

	**SS**	**HS**	**PI**	**CF**	**BA**	**BD**	**MIND**
SS	–						
HS	−0.165^**^	–					
PI	−0.273^**^	**0.393** ^**^	–				
CF	−0.221^**^	0.364^**^	**0.825** ^**^	–			
BA	0.126^**^	−0.012	−0.003	0.064	–		
BD	−0.378^**^	0.329^**^	**0.553** ^**^	**0.506** ^**^	−0.003	–	
MIND	0.288^**^	−0.365^**^	**−0.463** ^**^	**−0.460** ^**^	0.053	**−0.490** ^**^	–

^**^The correlation is significant for *p* < 0.01 (bilateral). ^*^The correlation is significant for *p* < 0.05 (bilateral). SS, sexual satisfaction; HS, hypersexuality; PI, psychological inflexibility; CF, cognitive fusion; BA, body awareness; BD, bodily dissociation; MIND, mindfulness skills. Moderate and high significant correlations are highlighted in bold.

As for the linear regression for the dependent variable hypersexuality, it emerged from the analysis that PI is the variable with the most weight (β = −0.285, *p* < 0.001). As for the linear regression for the dependent variable sexual satisfaction, it can be concluded that BD is the variable with the greatest weight (β = −0.311, *p* < 0.001).

## 4. Discussion

This study has explored the sociodemographic characteristics associated with hypersexuality and the role of some relevant variables in sexual satisfaction and hypersexuality in a Spanish sample. The level of hypersexuality was similar to the one in a previous study in Spain (Ballester-Arnal et al., 2019). The likelihood of hypersexuality was higher in males, lesbians and gay men, singles, and students. It could be hypothesized that the increased accessibility of sexual activities for single participants and students may contribute to the risk of hypersexuality. The higher prevalence of hypersexuality based on gender, sexual orientation, and relationship status is consistent with previous studies (Daneback et al., 2005; Ballester-Arnal et al., 2013; Castro-Calvo et al., 2017; Bothe et al., 2018; Slavin et al., 2020). Differences linked to the male gender may have been attributed to gender-differentiated informal sex education. As it has been stated before (Bothe et al., 2018), the higher prevalence may be attributed to stigmatization linked to minority sexual orientation so that sex activities can act as an effective way to feel accepted or reduce the discomfort generated by a homophobic environment (Silveira and Cerqueira-Santos, 2019). In addition, gay males could experience more challenges than heterosexual men when it comes to dating and forming romantic relationships so that sex without commitment becomes easier (Bothe et al., 2018). As for educational level, most participants with hypersexuality had university studies. Previous research (Kafka and Hennen, 1999) has related hypersexuality with lower educational levels. It is plausible that these results in the current investigation are due to a sampling bias given the university context in which it was conducted.

On the other hand, hypersexuality was positively correlated with PI, CF, and BD and negatively with mindfulness, with being PI the variable with the greatest weight. Sexual satisfaction showed a correlation with lower levels of hypersexuality, PI, CF, and BD, and with higher levels of BA and mindfulness, being BD the variable with the greatest weight. Regarding mindfulness, it has already been found to be a protective factor for compulsive sexual behavior (Reid et al., 2014b; Shorey et al., 2016). Beyond that, recent studies have revealed that mindfulness is related to reductions in sexual stigma in gay and bisexual men (Salvati et al., 2019), as well as in lesbian and bisexual women (Salvati and Chiorri, 2023). It can be hypothesized that this could be an additional way in which mindfulness may have a mediating role in reducing the risk of hypersexuality in LGBT people.

This study highlights the relevance that PI has as a variable on sexual satisfaction and, especially, on hypersexuality. Previous studies have pointed out a negative association between hypersexuality and anxiety (García-Barba et al., 2020), life satisfaction, emotional dysregulation, and loneliness (Dhuffar et al., 2015) and suggested its role in mood and stress regulation (Wordecha et al., 2018). Accordingly, prior studies have already suggested a relationship between hypersexuality and poor emotional regulation strategies and have stated compulsive sexual behavior may represent a delusive form of self-regulation and coping with distressing emotions (Goodman, 2001; Guigliamo, 2006; Cashwell et al., 2017; Lew-Starowicz et al., 2020). Unlike other studies that associate emotional dysregulation with the experience of child sexual abuse and insecure attachment patterns (Garofalo et al., 2016; Lew-Starowicz et al., 2020), this study focuses specifically on the role of emotional regulation through PI. Although sexual behavior can be very rewarding at first, due to physical pleasure, and positively reinforced, sexual compulsive behavior may be later negatively reinforced when it reduces negative mood associated with sadness, anxiety, or interpersonal stress (Cooper et al., 2000). In this regard, this study provides evidence in support of the hypothesis that sees impulsive sexual behaviors as avoidance behaviors that are directed toward reducing not only sexual impulse but also emotional negative states. In other words, hypersexuality would be explained by the use of sex as an emotional regulation strategy, which would be part of a larger pattern of experiential avoidance, or part of a larger pattern of inflexibility that in the long term would prove maladaptive (Hayes et al., 1996). This hypothesis could explain the relationship between negative emotions and hypersexuality found in previous studies (Dhuffar et al., 2015; García-Barba et al., 2020), so that compulsive sexual practices could be aimed at short-term control of the discomfort, despite often entailing a high cost on the physical and psychological health of the person. According to this hypothesis, the cause of hypersexuality would not be anxiety or negative mood itself, but rather the way of interacting with it, aimed toward avoidance. Thus, IP could explain how sexual behavior can evolve from healthy and non-problematic to compulsive and problematic (Wetterneck et al., 2012). On the other hand, cognitive fusion, one of the six processes which conform PI, has been shown to be related to hypersexuality and sexual dissatisfaction. This could highlight how problematic excessive adherence to verbal rules can be, as is shown, for example, when a person with compulsive sexual behaviors gets stuck in thoughts such as “I need to have sex and distract myself”, or when a person who is practicing sex gets trapped by thoughts such as “I need to be good enough”. These examples of cognitive fusion may illustrate how easily the person can get away from the present moment, focusing on the immediate consequences of avoidance behaviors but not considering the long-term cost, and the way verbalizations broaden and intensify the scope of threats (Blackledge, 2015). Therefore, this study provides justification for interventions specifically directed toward promoting PF and cognitive defusion, such as Acceptance and Commitment Therapy (ACT) (Hayes et al., 2011). In fact, this therapy has already begun to show its effectiveness in improving sexual satisfaction (Honarparvaran et al., 2010; Nezhad and Shameli, 2017), reducing time consumption of pornography (Crosby, 2011; Crosby and Twohig, 2016), and reducing hypersexuality (Lantheaume, 2018).

Moreover, this research also points out the importance of training mindfulness skills and reducing body dissociation for sexual satisfaction. Previous studies have already highlighted the need to take into account the role of mindfulness skills on sexual satisfaction improvement (Brotto and Heiman, 2007; Brotto and Basson, 2014; Selice and Morris, 2021). The results found in this research support the use of mindfulness-based psychological interventions aimed at increasing sexual satisfaction. Likewise, and in line with previous results (Lantheaume, 2018), the present study supports the need to consider mindfulness, as well as dealing with BD, in the treatment of hypersexuality. Mindfulness represents the ability to be present and paying attention to experience as it unfolds moment by moment (Kabat-Zinn, 2016). Given that sexual satisfaction is related to the ability to enjoy both physical pleasure and the interaction with the other person, the abilities that are developed through the practice of mindfulness can contribute toward people being more centered on the sexual experience and thus being able to experience it in a more pleasurable and satisfactory way. Low levels of body awareness have been associated with low sexual desire and excitation (Carvalheira et al., 2017). It seems clear that the further a person is disconnected from their sensations (and other bodily experiences) the less that person will enjoy the sexual encounter, just like sex therapy pioneers Masters and Johnson (1966) had pointed out with the “spectator role”. They defined this role as the hyper-focus on self-performance and physical appearance, which then gives place to the disconnection from sensations during sexual interactions.

Some limitations of the present investigation are listed next. Regarding the participants, it is worth pointing out that the sample was somewhat biased, having an over-representation of women, university students, supporters of left-wing politics, and heterosexuals. This could be attributed to the context where the data were obtained and the recruiting method. Hence, there is a need for more studies that meet the aims of this study while using a more diverse and representative sample to achieve more reliable results. In addition, since the data come exclusively from self-reports, it entails a degree of participant subjectivity and, given the low level of control over the conditions in which the questionnaire was answered, some responses could be biased. However, having obtained the data anonymously through the Internet could be beneficial with regard to the truthfulness of the answers given that it was such a sensible topic (Griffiths, 2012). In any case, addressing the issue of subjectivity should be considered for further research.

In conclusion, and despite the previously mentioned limitations, this study shows the increased vulnerability of lesbians and gay men, males, and singles to engaging in hypersexual behavior. It also justifies the consideration of psychological interventions based on PF when dealing with hypersexuality and highlights the need of taking into account mindfulness skills to increase sexual satisfaction. Future studies based on these results could extend scientific knowledge about this issue and, in particular, emphasize the importance of considering BD and mindfulness in the improvement of sexual satisfaction, as well as mindfulness and PF in more effective management of hypersexual behaviors.

## Data availability statement

The original contributions presented in the study are included in the article/supplementary material, further inquiries can be directed to the corresponding author.

## Ethics statement

The studies involving human participants were reviewed and approved by the Comité de ética de la Universidad Europea de Madrid. The patients/participants provided their written informed consent to participate in this study.

## Author contributions

All authors listed have made a substantial, direct, and intellectual contribution to the study and approved it for publication.

## References

[B1] Amamou B. Missaoui C. Haouala A. Ben M. A. Zaafrane F. Gaha L. (2020). Sexual addiction, self-esteem and personality dimensions. J. Addict. Ther. Res. 4, 6–12. 10.29328/journal.jatr.1001011

[B2] Ashdown B. K. Hackathorn J. Clark E. M. (2011). In and out of the bedroom: Sexual satisfaction in the marital relationship. J. Int. Soc. Sci. 2, 40–57. Available online at: https://www.jiss.org/documents/volume_2/issue_1/JISS_2011_Sexual_Satisfaction_in_Marriage.pdf

[B3] Ballester-Arnal R. Castro-Calvo J. Gil-Julia B. Giménez-García C. Gil-Llario M. D. (2019). A validation study of the spanish version of the hypersexual behavior inventory (HBI): paper-and-pencil versus online administration. J. Sex Marital Ther. 45, 283–302. 10.1080/0092623X.2018.1518886 30657020

[B4] Ballester-Arnal R. Gómez-Martínez S. Llario M. D. G. Salmerón-Sánchez P. (2013). Sexual compulsivity scale: adaptation and validation in the spanish population. J. Sex Marital Ther. 39, 526–540. 10.1080/0092623X.2012.665816 23631692

[B5] Barney J. L. (2023). Exploring Body Image Related Cognitive Fusion as a Maintenance Mechanism of Eating Disorder Pathology. Doctoral dissertation. Available online at: https://digitalcommons.usu.edu/cgi/viewcontent.cgi?article=9979&context=etd

[B6] Barraca J. (2004). Spanish adaptation of the acceptance and action questionnaire (AAQ). Int. J. Psychol. Ther. 4, 505–515.

[B7] Blackledge J. T. (2015). Cognitive Defusion in Practice: A Clinician's Guide to Assessing, Observing, and Supporting Change in Your Client. London: New Harbinger Publications.

[B8] Bond F. W. Hayes S. C. Baer R. A. Carpenter K. M. Guenole N. Orcutt H. K.. (2011). Preliminary psychometric properties of the acceptance and action questionnaire-II: a revised measure of psychological inflexibility and experiential avoidance. Behav. Ther. 42, 676–688. 10.1016/j.beth.2011.03.007 22035996

[B9] Bothe B. Bartók R. Tóth-Király I. Reid R. C. Griffiths M. D. Demetrovics Z.. (2018). Hypersexuality, gender, and sexual orientation: A large-scale psychometric survey study. Arch. Sex. Behav. 47, 2265–2276. 10.1007/s10508-018-1201-z 29926261

[B10] Bothe B. Kovács M. Tóth-Király I. Reid R. C. Griffiths M. D. Orosz G.. (2019). The psychometric properties of the hypersexual behavior inventory using a large-scale nonclinical sample. J. Sex Res. 56, 180–190. 10.1080/00224499.2018.1494262 30028633

[B11] Brotto L. A. Basson R. (2014). Group mindfulness-based therapy significantly improves sexual desire in women. Behav. Res. Ther. 57, 43–54. 10.1016/j.brat.2014.04.001 24814472

[B12] Brotto L. A. Heiman J. R. (2007). Mindfulness in sex therapy: applications for women with sexual difficulties following gynecologic cancer. Sex. Relation. Ther. 22, 3–11. 10.1080/14681990601153298

[B13] Brown K. W. Ryan R. M. (2003). The benefits of being present: mindfulness and its role in psychological well-being. J. Pers. Soc. Psychol. 84, 822–848. 10.1037/0022-3514.84.4.822 12703651

[B14] Byers E. S. (2005). Relationship satisfaction and sexual satisfaction: a longitudinal study of individuals in long-term relationships. The J. Sex Res. 42, 113–118. 10.1080/00224490509552264 16123841

[B15] Carvalheira A. Price C. Neves C. F. (2017). Body awareness and bodily dissociation among those with and without sexual difficulties: differentiation using the scale of body connection. J. Sex Marital Ther. 43, 801–810. 10.1080/0092623X.2017.1299823 28368683

[B16] Cashwell C. S. Giordano A. L. King K. Lankford C. Henson R. K. (2017). Emotion regulation and sex addiction among college students. Int. J. Mental Health Addict. 15, 16–27. 10.1007/s11469-016-9646-6

[B17] Castro-Calvo J. Ballester-Arnal R. Giménez-García C. Gil-Juliá B. (2017). Comportamiento sexual online en adultos mayores. Int. J. Dev. Educ. Psychol. 2, 89–98. 10.17060/ijodaep.2017.n2.v2.1082

[B18] Castro-Calvo J. Gil-Llario M. D. Giménez-García C. Gil-Juliá B. Ballester-Arnal R. (2020). Occurrence and clinical characteristics of Compulsive Sexual Behavior Disorder (CSBD): a cluster analysis in two independent community samples. J. Behav. Addict. 9, 446–468. 10.1556/2006.2020.00025 32554840 PMC8939411

[B19] Chatzittofis A. Savard J. Arver S. Öberg K. G. Hallberg J. Nordström P.. (2017). Interpersonal violence, early life adversity, and suicidal behavior in hypersexual men. J. Behav. Addict. 6, 187–193. 10.1556/2006.6.2017.027 28467102 PMC5520124

[B20] Cooper A. Delmonico D. L. Burg R. (2000). Cybersex users, abusers, and compulsives: new findings and implications. Sex. Addict. Compuls. J. Treatment Prev. 7, 5–29. 10.1080/10720160008400205

[B21] Cox D. W. Motl T. C. Bakker A. M. Lunt R. A. (2018). Cognitive fusion and post-trauma functioning in veterans: examining the mediating roles of emotion dysregulation. J. Cont. Behav. Sci. 8, 1–7. 10.1016/j.jcbs.2018.02.002

[B22] Crosby J. M. (2011). Acceptance and Commitment Therapy for the Treatment of Compulsive Pornography Use: A Randomized Clinical Trial. Utah: Utah State University.

[B23] Crosby J. M. Twohig M. P. (2016). Acceptance and commitment therapy for problematic internet pornography use: a randomized trial. Behav. Ther. 47, 355–366. 10.1016/j.beth.2016.02.001 27157029

[B24] Daneback K. Cooper A. Månsson S. A. (2005). An internet study of cybersex participants. Arch. Sex. Behav. 34, 321–328. 10.1007/s10508-005-3120-z 15971014

[B25] Dhuffar M. K. Pontes H. M. Griffiths M. D. (2015). The role of negative mood states and consequences of hypersexual behaviours in predicting hypersexuality among university students. J. Behav. Addict. 4, 181–188. 10.1556/2006.4.2015.030 26551908 PMC4627679

[B26] Dove N. L. Wiederman M. W. (2000). Cognitive distraction and women' s sexual functioning. J. Sex Marital Ther. 26, 67–78. 10.1080/009262300278650 10693117

[B27] Dwulit A. D. Rzymski P. (2019). The potential associations of pornography use with sexual dysfunctions: an integrative literature review of observational studies. J. Clin. Med. 8, 1–15. 10.3390/jcm8070914 31247949 PMC6679165

[B28] Dyar C. Newcomb M. E. Mustanski B. Whitton S. W. (2020). A structural equation model of sexual satisfaction and relationship functioning among sexual and gender minority individuals assigned female at birth in diverse relationships. Archi. Sex. Behav. 49, 693–710. 10.1007/s10508-019-1403-z 31407194 PMC7012718

[B29] García-Barba M. Ballester-Arnal R. Gil-Llario M. D. Castro-Calvo J. Nebot-García J. E. (2020). El papel de los problemas emocionales en la hipersexualidad. Int. J. Dev. Educ. Psychol. 1, 443–452. 10.17060/ijodaep.2020.n1.v1.1806

[B30] Garofalo C. Velotti P. Zavattini G. C. (2016). Emotion dysregulation and hypersexuality: Review and clinical implications. Sexual Relat. Ther. 31, 3–19. 10.1080/14681994.2015.1062855

[B31] Gillanders D. T. Bolderston H. Bond F. W. Dempster M. Flaxman P. E. Campbell L.. (2014). The development and initial validation of the cognitive fusion questionnaire. Behav. Ther. 45, 83–101. 10.1016/j.beth.2013.09.001 24411117

[B32] Gloster A. T. Klotsche J. Chaker S. Hummel K. V. Hoyer J. (2011). Assessing psychological flexibility: What does it add above and beyond existing constructs?. Psychol. Assess. 23, 970. 10.1037/a0024135 21767025

[B33] Goodman A. (2001). What's in a name? terminology for designating a syndrome of driven sexual behavior. Sexual Addicti. Compul. 8, 191–213. 10.1080/107201601753459919

[B34] Griffiths M. D. (2012). The use of online methodologies in studying paraphilias - a review. J. Behav. Addict. 1, 143–150. 10.1556/JBA.1.2012.4.1 26165601

[B35] Grov C. Parsons J. T. Bimbi D. S. (2010). Sexual compulsivity and sexual risk in gay and bisexual men. Arch. Sexual Behav. 39, 940–949. 10.1007/s10508-009-9483-9 19308715 PMC2890042

[B36] Guigliamo J. (2006). Out of control sexual behavior: a qualitative investigation. Sex. Addict. Compul. 13, 361–375. 10.1080/10720160601011273

[B37] Hajivosough N. S. Tavakolizadeh J. Rajayi A. Atarodi A. (2012). The effect of cognitive-behavioral teaching on improvement of women's sexual function with hypoactive sexual desire disorder. Zahedan J. Res. Med. Scie. 14, 100–102.

[B38] Hayes S. (2019). A Liberated Mind: The Essential Guide to ACT. London: Random House.

[B39] Hayes S. C. (2020). Una mente liberada. La Guia Esencial de la Terapia de Aceptación y Compromiso (ACT). Bern: Paidós.

[B40] Hayes S. C. Levin M. E. Plumb-Vilardaga J. Villatte J. L. Pistorello J. (2013). Acceptance and commitment therapy and contextual behavioral science: Examining the progress of a distinctive model of behavioral and cognitive therapy. Behav. Ther. 44, 180–198. 10.1016/j.beth.2009.08.002 23611068 PMC3635495

[B41] Hayes S. C. Luoma J. B. Bond F. W. Masuda A. Lillis J. (2006). Acceptance and commitment therapy: Model, processes and outcomes. Behav. Res. Ther. 44, 1–25. 10.1016/j.brat.2005.06.006 16300724

[B42] Hayes S. C. Strosahl K. D. Wilson K. G. (2011). Acceptance and Commitment Therapy: The Process and Practice of Mindful Change. Guilford Press.26321980

[B43] Hayes S. C. Wilson K. G. Gifford E. V. Follette V. M. Strosahl K. (1996). Experiential avoidance and behavioral disorders: a functional dimensional approach to diagnosis and treatment. J. Consult. Clin. Psychol. 64, 1152–1168. 10.1037/0022-006X.64.6.1152 8991302

[B44] Higgins J. A. Mullinax M. Trussell J. Kenneth Davidson J. Moore N. B. (2011). Sexual satisfaction and sexual health among university students in the United States. Am. J. Publ. Health 101, 1643–1654. 10.2105/AJPH.2011.300154 21778509 PMC3154236

[B45] Honarparvaran N. Tabrizy M. Navabinejad S. Shafiabady A. Moradi M. (2010). The efficacy of acceptance and commitment therapy (ACT) training with regard to reducing sexual dissatisfaction among couples. Eur. J. Soc. Sci. 15, 166–172.

[B46] Hsieh K. Y. Hsiao R. C. Yang Y. H. Lee K. H. Yen C. F. (2019). Relationship between self-identity confusion and internet addiction among college students: the mediating effects of psychological inflexibility and experiential avoidance. Int. J. Environ. Res. Publ. Health 16, 3225. 10.3390/ijerph16173225 31484435 PMC6747481

[B47] Jo D. Yang E. (2019). The role of present moment awareness and cognitive fusion with food craving in the relationship between depression and binge eating. J. Cont. Behav. Sci 13, 126–133. 10.1016/j.jcbs.2019.08.001

[B48] Kabat-Zinn J. (2016). Vivir con plenitud las crisis. Cómo utilizar la sabiduría del cuerpo y de la mente para enfrentarnos al estrés, al dolor y la enfermedad. Kairós.

[B49] Kafka M. P. (2010). Hypersexual disorder: a proposed diagnosis for DSM-V. Arch. Sexual Behav. 39, 377–400. 10.1007/s10508-009-9574-7 19937105

[B50] Kafka M. P. Hennen J. (1999). The paraphilia-related disorders: An empirical investigation of nonparaphilic hypersexuality disorders in outpatient males. J. Sex Marital Ther. 25, 305–319. 10.1080/00926239908404008 10546169

[B51] Klein V. Rettenberger M. Briken P. (2014). Self-reported indicators of hypersexuality and its correlates in a female online sample. J. Sex. Med. 11, 1974–1981. 10.1111/jsm.12602 24909396

[B52] Kollman D. M. Brown T. A. Barlow D. H. (2009). The construct validity of acceptance: a multitrait–multimethod investigation. Behav. Ther. 40, 205–218. 10.1016/j.beth.2008.06.002 19647522

[B53] Kowalewska E. Kraus S. W. Lew-Starowicz M. Gustavsson K. Gola M. (2019). Which dimensions of human sexuality are related to compulsive sexual behavior disorder (CSBD)? Study using a multidimensional sexuality questionnaire on a sample of polish males. J. Sexual Med. 16, 1264–1273. 10.1016/j.jsxm.2019.05.006 31204265

[B54] Kuru T. Celenk S. (2021). The relationship among anxiety, depression, and problematic smartphone use in university students: the mediating effect of psychological inflexibility. Alpha Psychiatry 22, 10–5455. 10.5455/apd.136695 36425443 PMC9590678

[B55] Lantheaume S. (2018). Thérapie d'acceptation et d'engagement (ACT) et addiction sexuelle. Encephale 44, 387–391. 10.1016/j.encep.2017.06.001 28870687

[B56] Lawrance K. A. Byers E. S. (1995). Sexual satisfaction in long-term heterosexual relationships: The interpersonal exchange model of sexual satisfaction. Pers. Relationships 2, 267–285. 10.1111/j.1475-6811.1995.tb00092.x27077235

[B57] Lew-Starowicz M. Lewczuk K. Nowakowska I. Kraus S. Gola M. (2020). Compulsive sexual behavior and dysregulation of emotion. Sexual Med. Rev. 8, 191–205. 10.1016/j.sxmr.2019.10.003 31813820

[B58] Liu C. Rotaru K. Chamberlain S. R. Ren L. Fontenelle L. F. Lee R. S.. (2022). The moderating role of psychological flexibility on the association between distress-driven impulsivity and problematic internet use. Int J. Environ. Res. Public Health 19, 9592. 10.3390/ijerph19159592 35954947 PMC9368545

[B59] Masters W. H. Johnson V. E. (1966). Human Sexual Response. London: Little, Brown and Company.

[B60] Mayland K. A. (2005). The impact of practicing mindfulness meditation on women's sexual lives [Thesis, The California School of Professional Psychology].

[B61] Mehling W. E. Gopisetty V. Daubenmier J. Price C. J. Hecht F. M. Stewart A.. (2009). Body awareness: construct and self-report measures. PloS ONE 4, e5614. 10.1371/journal.pone.0005614 19440300 PMC2680990

[B62] Miner M. H. Dickenson J. Coleman E. (2019). Effects of Emotions on Sexual Behavior in Men with and without Hypersexuality. PSex Addict Compulsivity 26, 24–41. 10.1080/10720162.2018.1564408 31772491 PMC6879181

[B63] Nezhad M. S. Shameli L. (2017). The effect of acceptance and commitment therapy on sexual satisfaction of couples in Shiraz. Int. J. Medical Res. Health Sci. 6, 58–65.

[B64] Ong C. W. Pierce B. G. Petersen J. M. Barney J. L. Fruge J. E. Levin M. E.. (2020). A psychometric comparison of psychological inflexibility measures: discriminant validity and item performance. J. Contextual Behav. Sci. 18, 34–47. 10.1016/j.jcbs.2020.08.007

[B65] Páez M. Montesinos F. (2019). Claves y evidencias en la aplicación de la terapia de aceptación y compromiso en el tratamiento de las adicciones. Análisis y Modificación de Conducta 45, 171–172. 10.33776/amc.v45i171-2.3682

[B66] Price C. J. Thompson E. A. (2007). Measuring dimensions of body connection: Body awareness and bodily dissociation. J. Alternat. Compl. Med. 13, 945–953. 10.1089/acm.2007.0537 18047441 PMC3029599

[B67] Quezada-Berumen L. D. C. González-Ramírez M. T. Cebolla A. Soler J. Garcla-Campayo J. (2014). Body awareness and mindfulness: validation of the Spanish version of the Scale of Body Connection. Actas Espanolas de Psiquiatria 42, 57–67. 24715363

[B68] Reid R. C. Bramen J. E. Anderson A. Cohen M. S. (2014a). Mindfulness, emotional dysregulation, impulsivity, and stress proneness among hypersexual patients. J. Clin. Psychol. 70, 313–321. 10.1002/jclp.22027 23852856

[B69] Reid R. C. Carpenter B. N. Draper E. D. Manning J. C. (2010). Exploring psychopathology, personality traits, and marital distress among women married to hypersexual men. J. Couple Relationship Ther. 9, 203–222. 10.1080/15332691.2010.491782

[B70] Reid R. C. Garos S. Carpenter B. N. (2011). Reliability, validity, and psychometric development of the hypersexual behavior inventory in an outpatient sample of men. Sexual Addict. Compul. 18, 30–51. 10.1080/10720162.2011.555709

[B71] Reid R. C. Temko J. Moghaddam J. F. Fong T. W. (2014b). Shame, rumination, and self-compassion in men assessed for hypersexual disorder. J. Psychiatr. Prac. 20, 260–268. 10.1097/01.pra.0000452562.98286.c5 25036581

[B72] Romero-Moreno R. Márquez-González M. Losada A. Gillanders D. Fernández-Fernández V. (2014). Cognitive fusion in dementia caregiving: Psychometric properties of the Spanish version of the Cognitive Fusion Questionnaire. Behav. Psychol. 22, 117–132.

[B73] Ruiz F. J. Langer Herrera Á. I. Luciano C. Cangas A. J. Beltrán I. (2013). Measuring experiential avoidance and psychological inflexibility: the Spanish version of the acceptance and action questionnaire - II. Psicothema 25, 123–129. 10.1037/t65412-000 23336554

[B74] Salvati M. Chiorri C. (2023). Dispositional mindfulness in heterosexual and lesbian/bisexual women: associations with sexual prejudice and internalized sexual stigma. J. Homosexuality 70, 448–472. 10.1080/00918369.2021.1990686 34651561

[B75] Salvati M. Chiorri C. Baiocco R. (2019). The relationships of dispositional mindfulness with sexual prejudice and internalized sexual stigma among heterosexual and gay/bisexual men. Mindfulness 10, 2375–2384. 10.1007/s12671-019-01215-6

[B76] Sánchez-Sánchez L. C. Rodríguez M. F. V. García-Montes J. M. Petisco-Rodríguez C. Fernández-García R. (2021). Mindfulness in sexual activity, sexual satisfaction and erotic fantasies in a non-clinical sample. Int. J. Environ. Res. Public Health 18, 1161. 10.3390/ijerph18031161 33525611 PMC7908537

[B77] Santos-Iglesias P. Sierra J. C. (2010). El papel de la asertividad sexual en la sexualidad humana: una revisión sistemática. Int. J. Clin. Health Psychol. 10, 553–577. Available online at: http://www.aepc.es/ijchp/articulos_pdf/ijchp-366.pdf

[B78] Selice L. Morris K. L. (2021). Mindfulness and sexual dysfunction: a systematic research synthesis. J. Sex Marital Therapy 5, 1–20. 10.1080/0092623X.2021.1991536 34693896

[B79] Shorey R. C. Elmquist J. Gawrysiak M. J. Anderson S. Stuart G. L. (2016). The relationship between mindfulness and compulsive sexual behavior in a sample of men in treatment for substance use disorders. Mindfulness 7, 866–873. 10.1007/s12671-016-0525-9 27570568 PMC4996480

[B80] Silveira A. P. Cerqueira-Santos E. (2019). Homofobia internalizada y satisfaccion sexual en parejas homosexuales. Psicogente 22, 307. 10.17081/psico.22.41.3307

[B81] Slavin M. N. Blycker G. R. Potenza M. N. Bothe B. Demetrovics Z. Kraus S. W.. (2020). Gender-related differences in associations between sexual abuse and hypersexuality. The J. Sexual Med. 17, 2029–2038. 10.1016/j.jsxm.2020.07.008 32792283 PMC7875089

[B82] Soler J. Tejedor R. Feliu-Sol A. Pascual J. C. Cebolla A. Soriano J.. (2012). Propiedades psicométricas de la versión española de la escala Mindful Attention Awareness Scale (MAAS). Actas Españolas de Psiquiatría 40, 18–25. 10.1037/t86572-000 22344492

[B83] Spenhoff M. Kruger T. H. C. Hartmann U. Kobs J. (2013). Hypersexual behavior in an online sample of males: associations with personal distress and functional impairment. J. Sexual Med. 10, 2996–3005. 10.1111/jsm.12160 23578375

[B84] Strizzi J. Fernández-Agis I. Alarcón-Rodríguez R. Parrón-Carreño T. (2016). Adaptation of the new sexual satisfaction scale-short form into Spanish. J. Sex Marit. Ther. 42, 579–588. 10.1080/0092623X.2015.1113580 26643768

[B85] Štulhofer A. Buško V. Brouillard P. (2010). Development and bicultural validation of the new sexual satisfaction scale. J. Sex Res. 47, 257–268. 10.1080/00224490903100561 19629836

[B86] Štulhofer A. Jurin T. Briken P. (2016). Is high sexual desire a facet of male hypersexuality? Results from an online study. J. Sex Mari. Ther. 42, 665–680. 10.1080/0092623X.2015.1113585 26569650

[B87] Tapp R. L. (2014). Concurrent Relations Between Psychological Flexibility and Sexual Satisfaction Among Committed Couples. Spalding: Spalding University.

[B88] Terzera L. Rimoldi S. M. L. Barbiano di Belgiojoso E. (2020). Sexual satisfaction among italian university students. Genus 76, 1–17. 10.1186/s41118-020-00096-1

[B89] van Kolthoorn A. M. (2018). The relationship between positive body image and sexual satisfaction in women: The role of body awareness and subjective sexual arousal (Master's thesis). Available online at: https://studenttheses.uu.nl/handle/20.500.12932/30315

[B90] Ventegodt S. (1998). Sex and the quality of life in Denmark. Arch. Sexual Behav. 27, 295–307. 10.1023/A:1018655219133 9604118

[B91] Wéry A. Billieux J. (2016). Online sexual activities: an exploratory study of problematic and non-problematic usage patterns in a sample of men. Comput. Hum. Behav. 56, 257–266. 10.1016/j.chb.2015.11.046

[B92] Wetterneck C. T. Burgess A. J. Short M. B. Smith A. H. Cervantes M. E. (2012). The role of sexual compulsivity, impulsivity, and experiential avoidance in internet pornography use. Psychol. Record 62, 3–18. 10.1007/BF03395783

[B93] Wordecha M. Wilk M. Kowalewska E. Skorko M. Łapiński A. Gola M.. (2018). “Pornographic binges” as a key characteristic of males seeking treatment for compulsive sexual behaviors: Qualitative and quantitative 10-week-long diary assessment. J. Beha. Addictions 7, 433–444. 10.1556/2006.7.2018.33 29865868 PMC6174597

[B94] World Health Organization. (2018). ICD-11 for Mortality and Morbidity Statistics. Available online at: https://icd.who.int/browse11/l-m/en (accessed June 22, 2018).

[B95] Yoo H. Bartle-Haring S. Day R. D. Gangamma R. (2014). Couple communication, emotional and sexual intimacy, and relationship satisfaction. J. Sex Marital Ther. 40, 275–293. 10.1080/0092623X.2012.751072 24111536

[B96] Zeliha T. R. A. S. (2022). Examining the relationships between psychological inflexibility and impulsiveness of university students. J. Career Review 1, 1–10. 10.5281/zenodo.7492590

